# Two Very Long Chain Fatty Acid Acyl-CoA Synthetase Genes, *acs-20* and *acs-22*, Have Roles in the Cuticle Surface Barrier in *Caenorhabditis elegans*

**DOI:** 10.1371/journal.pone.0008857

**Published:** 2010-01-25

**Authors:** Eriko Kage-Nakadai, Hiroyuki Kobuna, Masako Kimura, Keiko Gengyo-Ando, Takao Inoue, Hiroyuki Arai, Shohei Mitani

**Affiliations:** 1 Department of Physiology, Tokyo Women's Medical University School of Medicine, Tokyo, Japan; 2 Graduate School of Pharmaceutical Sciences, University of Tokyo, Tokyo, Japan; 3 Core Research for Evolutional Science and Technology (CREST), Japan Science and Technology Agency (JST), Saitama, Japan; New England Biolabs, United States of America

## Abstract

In multicellular organisms, the surface barrier is essential for maintaining the internal environment. In mammals, the barrier is the stratum corneum. Fatty acid transport protein 4 (FATP4) is a key factor involved in forming the stratum corneum barrier. Mice lacking *Fatp4* display early neonatal lethality with features such as tight, thick, and shiny skin, and a defective skin barrier. These symptoms are strikingly similar to those of a human skin disease called restrictive dermopathy. FATP4 is a member of the FATP family that possesses acyl-CoA synthetase activity for very long chain fatty acids. How *Fatp4* contributes to skin barrier function, however, remains to be elucidated. In the present study, we characterized two *Caenorhabditis elegans* genes, *acs-20* and *acs-22*, that are homologous to mammalian *FATP*s. Animals with mutant *acs-20* exhibited defects in the cuticle barrier, which normally prevents the penetration of small molecules. *acs-20* mutant animals also exhibited abnormalities in the cuticle structure, but not in epidermal cell fate or cell integrity. The *acs-22* mutants rarely showed a barrier defect, whereas *acs-20;acs-22* double mutants had severely disrupted barrier function. Moreover, the barrier defects of *acs-20* and *acs-20;acs-22* mutants were rescued by *acs-20*, *acs-22,* or human *Fatp4* transgenes. We further demonstrated that the incorporation of exogenous very long chain fatty acids into sphingomyelin was reduced in *acs-20* and *acs-22* mutants. These findings indicate that *C. elegans Fatp4* homologue(s) have a crucial role in the surface barrier function and this model might be useful for studying the fundamental molecular mechanisms underlying human skin barrier and relevant diseases.

## Introduction

The surface barrier is essential for maintaining the internal environment in multicellular organisms. The stratum corneum comprises this barrier in mammals [1], [2]. Fatty acid transport protein 4 (FATP4) is one of the key factors involved in forming the stratum corneum barrier [3], [4], [5] and was initially identified as a transport protein that facilitates the uptake of long chain fatty acids [6]. FATP also possesses acyl-coenzyme A (CoA) synthetase activity, which esterifies fatty acids into metabolically active CoA thioesters [7]. The mammalian FATP family comprises six members that differ in their biochemical activities, tissue expression patterns, and physiologic roles [8]. FATP1, 2, and 4 possess acyl-CoA synthetase activity towards very long chain fatty acids (VLCFAs) [9]. FATP5 is involved in metabolizing bile acids [10]. FATP3 and FATP6 were recently cloned and characterized, but their physiologic roles remain largely unclear [11], [12]. Several lines of evidence indicate the involvement of FATPs in some human diseases. FATP1 is the major FATP family member expressed in adipose tissue and in skeletal muscle [6] and *FATP1* knockout (KO) mice are completely resistant to diet-induced obesity, insulin desensitization, and other parameters of metabolic syndrome [13]. FATP4, one of the most well studied FATP members, is predominantly expressed in intestinal epithelial cells, and widely expressed in many organs including the skin [8]. Among the six family members, only FATP4 plays a crucial role in skin development and function. A mouse strain, *wrinkle-free* (*wrfr*), containing a spontaneous mutation that abrogates *Fatp4* expression, and *Fatp4*-KO mice display features of lethal dermatology, such as tight, thick, shiny skin and a defective skin barrier, and die shortly after birth, symptoms that are strikingly similar to those of a human skin disease called restrictive dermopathy [3], [4], [5]. How *Fatp4* contributes to the skin barrier function, however, is unknown.

A database search for sequences similar to human FATP members revealed homologues in various animals, including the simple multicellular animal, *Caenorhabditis elegans*, suggesting that FATP plays a fundamental role in animals [14]. Here we characterized two *C. elegans FATP* genes, *acs-20* and *acs-22*, and revealed that *C. elegans FATP*s are involved in forming the cuticle barrier and can be substituted for by human *Fatp4*.

## Results

### *C. elegans* FATP Family Members, *acs-20* and *acs-22,* Are Predominantly Expressed in the Hypodermis and Intestine, Respectively

**Figure 1:**
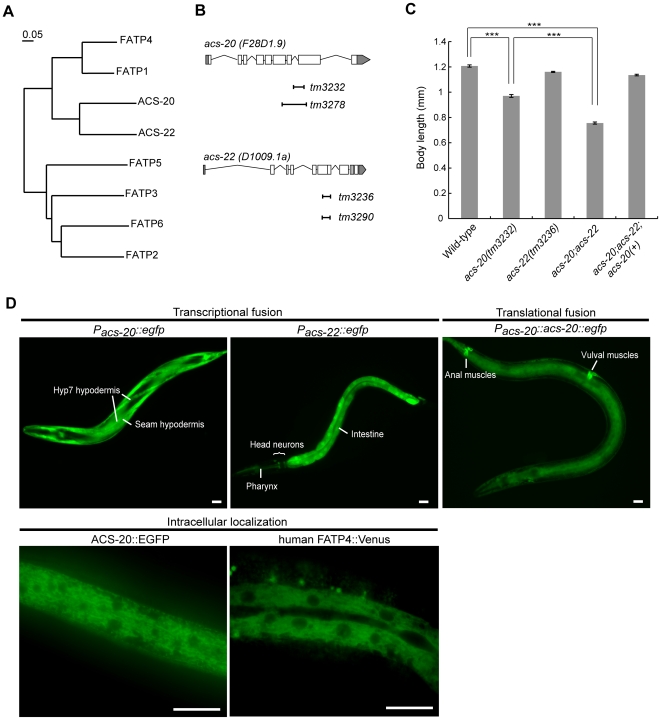
Deletion mutants and expression of *acs-20* and *acs-22.* (A) The phylogenic tree of FATPs among human and *C. elegans* was constructed using ClustalW (http://align.genome.jp). The sequences of a strongly conserved region corresponding to amino acids 246–556 in human FATP1 [14] were compared. The scale bar represents the evolutionary distance. (B) Schematic structures of genes and deletion mutations. (C) Body size of mutants and a rescue line. *acs-20 (tm3232); acs-22(tm3236);tmEx1920[P_acs-20_::acs-20::egfp]* was used as the rescue line. The body length of the animals (n = 50-64) was measured for each strain. Error bars indicate the mean ± SEM. ***P<0.001. (D) Expression patterns of transcriptional translational fusion genes. Photographed animals were L2-L3 stage larvae. ACS-20::EGFP and human FATP4::Venus are expressed in a reticular pattern throughout the cytoplasm in the hypodermis. Scale bar  = 20 µm.

We identified and cloned two genes in the *C. elegans* genome, *acs-20* (*F28D1.9*) and *acs-22* (*D1009.1a*), both of which are highly homologous to the mammalian FATP family. Phylogenic tree analysis of worm and human FATP family proteins showed that worm ACS-20 and ACS-22 are closely related to human FATP1 and FATP4, and rather distantly related to the other FATP family members FATP2, FATP3, FATP5, and FATP6 (Fig. 1A).

To investigate the roles of *acs-20* and *acs-22* in *C. elegans*, we isolated *acs-20* deletion alleles (*tm3232* and *tm3278*) and *acs-22* deletion alleles (*tm3236* and *tm3290*) from trimethylpsoralen/ultraviolet mutagenized libraries (Fig. 1B). *acs-20* mutants had a small body size and a slightly dumpyish phenotype, whereas *acs-22* mutants showed no apparent abnormal phenotypes (Fig. 1C). *acs-20;acs-22* double mutant animals were significantly smaller than *acs-20* mutant animals, suggesting that *acs-22* acts redundantly with *acs-20* (Fig. 1C).

To determine their expression patterns, we first performed expression analyses with transcriptional fusion genes. *P_acs-20_::egfp* was expressed predominantly in epithelial cells, such as Hyp7 and seam hypodermis (Fig.1D), while *P_acs-22_::egfp* was strongly expressed in the intestine, moderately expressed in the pharynx and in several sets of unidentified head neurons (Fig.1B and D), and faintly expressed in the hypodermis (data not shown). Translational fusion *P_acs-20_::acs-20::egfp* was expressed in the vulval and anal muscles, in addition to Hyp7 and seam hypodermis (Fig. 1C). The *P_acs-20_::acs-20::egfp* transgene (Fig. 1D), which was expressed in a reticular pattern throughout the cytoplasm (Fig. 1D, ACS-20::EGFP), fully rescued the reduced body size of the *acs-20;acs-22* double mutants (Fig. 1C), suggesting its functional expression in the endoplasmic reticulum (ER).

### *acs-20* Plays an Essential Role in Surface Barrier Function

**Figure 2:**
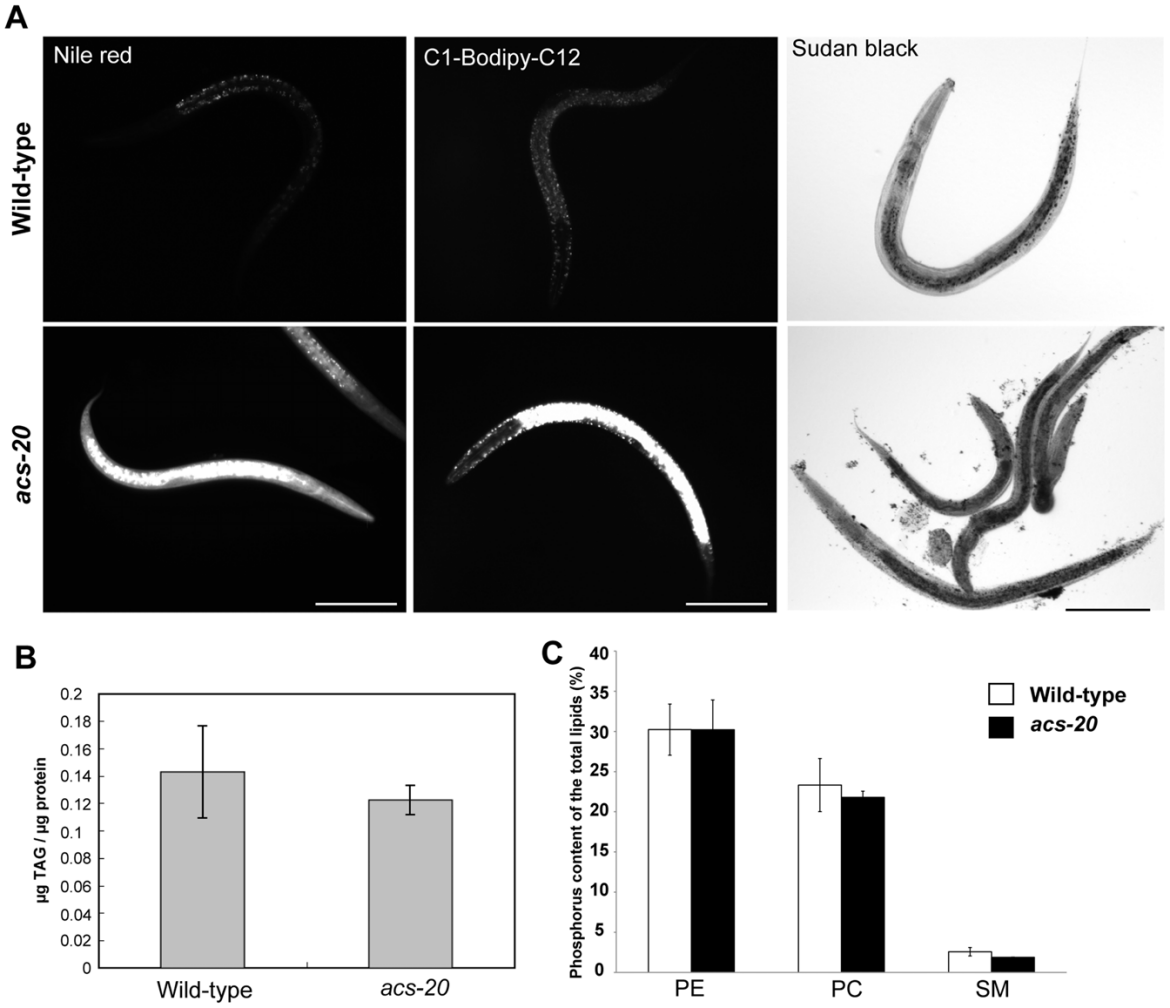
*acs-20* mutants are strongly stained with lipophilic dyes, but their lipid contents are not altered. (A) Wild-type (left panels) and *acs-20 (tm3232)* mutant (right panels) animals are stained with Nile Red, C1-Bodipy-C12, and Sudan black, respectively. Scale bars  = 100 µm. (B) Amounts of triacylglycerol in wild-type and *acs-20* mutant animals. (C) Lipid composition in wild-type and *acs-20* mutant animals. (B, C) Error bars indicate SEM of three measurements.

First, to determine whether *acs-20* and *acs-22* mutants affect lipid metabolism, we examined fat storage using the lipophilic fluorescent dye, Nile Red. *acs-20* mutant animals showed excessively high fluorescence intensity in the intestine (Fig. 2A), while *acs-22* mutant animals showed no obvious abnormal intensity (data not shown). Staining with another lipophilic fluorescent compound, C1-BODIPY-C12, produced similar results (Fig. 2A). On the other hand, Sudan black staining, which is used for general lipid staining but requires the samples to be permeabilized, exhibited no significant difference between wild-type and *acs-20* mutant animals (Fig. 2A, right). Thus, we directly quantified the triacylglycerol and phospholipid contents. There was no significant difference between wild-type and *acs-20* mutant animals (Fig. 2B, C). Based on these contradictory results, we considered the possibility that *acs-20* mutant animals have a hyper-permeable surface barrier rather than increased lipid accumulation.

**Figure 3:**
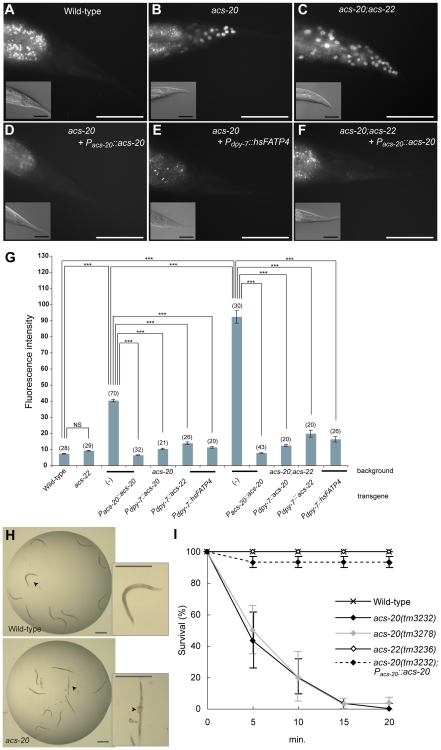
*acs-20* is required for surface barrier function against small molecule permeability and hypo-osmolality. (A–F) Permeability to Hoechst 33342 in wild-type (A), *acs-20 (tm3232)* (B), *acs-20(tm3232);acs-22(tm3236)* (C), *acs-20(tm3232);tmEx1995[P_acs-20_::acs-20:egfp]* (D), *acs-20(tm3232);tmEx2072[P_dpy-7_::hsFATP4::Venus]* (E), and *acs-20(tm3232);acs-22(tm3236);tmEx1920[P_acs-20_::acs-20::egfp]* (F). (Insets): Differential interference contrast images. Scale bars  = 50 µm. (G) Intensity of the Hoechst staining. *P_dpy-7_::acs-20*: *tmEx1955[P_dpy-7_::acs-20::Venus]*, *P_dpy-7_::acs-22*: *tmEx1983[P_dpy-7_::acs-22::Venus]*. The number of samples is indicated in parentheses. Error bars indicate the mean ± SEM. ***P<0.001. NS: Not Significant. (H) Bright-field images of wild-type (upper panels) and *acs-20 (tm3232)* (lower panels) worms soaked in water for 20 min. *acs-20* animals became rigid and some of them had burst vulvae. (Insets): Magnified photographs of animals indicated by arrows. Arrowhead indicates a burst vulva. Scale bars  = 0.5 mm. (I) Survival curves of wild-type, *acs-20 (tm3232)*, *acs-20 (tm3278)*, *acs-22 (tm3236)* mutants, and an *acs-20(tm3232);tmEx1995[P_acs-20_::acs-20::egfp]* rescue line in water. The number of surviving animals was counted at each time point and the survival rates are plotted. Error bars indicate SEMs of three measurements.

To examine the surface barrier function, living worms were incubated for 15 min in buffer containing a nuclei-staining dye Hoechst 33342, which does not usually permeate the worm cuticle barrier. The *acs-20* mutant had increased Hoechst permeability compared to wild-type (Fig. 3A, B, and G). In contrast, the majority of *acs-22* mutant animals did not have increased Hoechst permeability. The *acs-20;acs-22* double mutant showed more intense staining than the *acs-20* single mutant (Fig. 3B, C, and G), suggesting that *acs-22* also contributes to the surface barrier function. The impaired surface barrier of *acs-20* single mutants and *acs-20;acs-22* double mutants was fully rescued by the *P_acs-20_::acs-20::egfp* transgene (Fig. 2D, F, and G). The expression of *acs-20* or *acs-22* with the *dpy-7* promoter, which drives expression in hyp7 hypodermis restored the impaired barrier of *acs-20* and *acs-20;acs-22* mutants although not fully, indicating that *acs-20* functions in hyp7 hypodermis for the surface barrier and *acs-22* can substitute for *acs-20* when *acs-22* is expressed in hyp7 hypodermis with the *dpy-7* promoter. These findings are similar to those in studies of *Fatp4*, which is expressed and functions in the skin and *Fatp4*-KO mice exhibit epidermal barrier defects [3], [4], [5]. To test the idea that *acs-20* is a functional orthologue of mammalian *Fatp4*, we examined whether the expression of human *Fatp4* cDNA rescues the defective surface barrier of *acs-20* and *acs-20;acs-22* mutants. The *dpy-7* promoter-driven human FATP4 induced significant rescue activity (Fig. 3E and G), and in rescued strains, the human FATP4::Venus fusion protein was expressed in a reticular pattern (Fig. 1D, middle lower panel).

To further analyze their barrier function, we established an assay to measure the resistance to hypotonicity. When living worms were placed in a drop of H_2_O and incubated for 20 min, wild-type animals actively moved throughout the assay (Fig. 3H upper panels and 3I). As expected, *acs-20* animals were sensitive to hypotonicity: they gradually became rigid and some exhibited burst vulvae (Fig. 3H lower panels and 3I), probably due to the penetration of ambient H_2_O molecules into the worm coelom. The *acs-20* transgene fully rescued the defect of *acs-20* mutants (Fig. 3I). *acs-20* mutants were also hypersensitive to levamisole, an antiparasitic agent (data not shown). These results demonstrate that *acs-20* is required for barrier function, preventing the penetration of a variety of small molecules.

### *acs-20* Mutants Have Impaired Cuticle Structures

**Figure 4:**
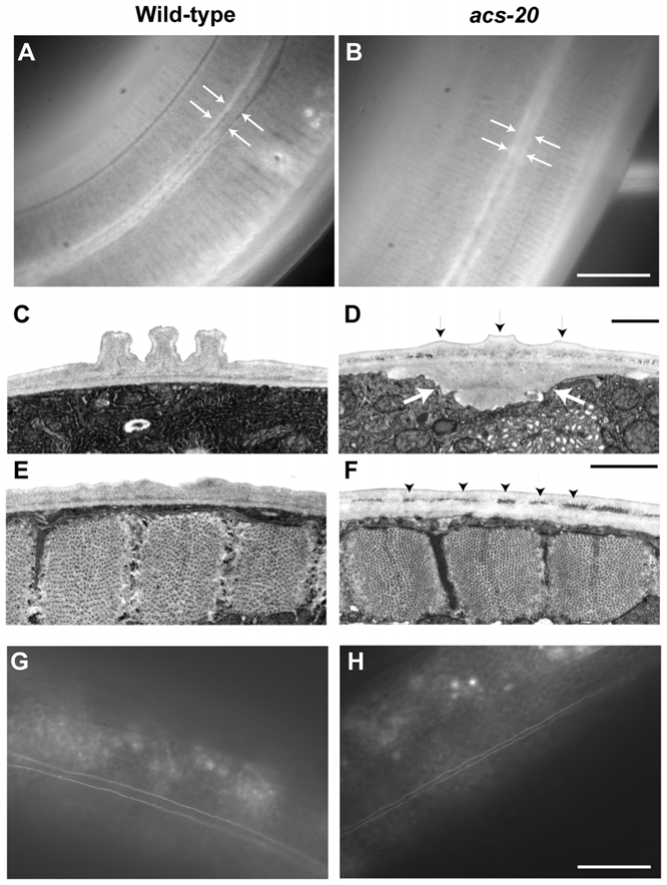
*acs-20* mutant exhibits various defects in the structures of the cuticle layer. (A, B) Localization of cuticle collagens. Fluorescent images of the localization of COL-19::GFP in wild-type (A) and in the *acs-20* (B) background. Arrows indicate alae. The *acs-20* animals display unclear alae ridges. Scale bar  = 20 µm. (C–F) Transmission electron micrographs of the outer layer of N2 (C, E) and *acs-20* (*tm3232*) (D, F). Black arrows indicate the incompletely extended ridges of the alae and white bold arrows indicate a pit, which probably contains unsecreted collagen (D). Arrowheads indicate unusual particles between the outer and inner cuticle layers (F). Scale bars  = 1 µm. (G, H) AJM-1::GFP localization in adult hermaphrodites of wild-type (G) and *acs-20* animals (H).

To determine the surface barrier structure and function of *acs-20* mutants, we first visualized the cuticle structure of the *acs-20* mutant with a *col-19::gfp* marker. COL-19::GFP was localized along the alae (white arrows in Fig. 4A) and annular furrows corresponding to the regular stripe pattern in the wild-type background. In the *acs-20* mutant background, although COL-19::GFP was localized correctly, the alae were obscured (white arrows in Fig. 4B).

To directly examine the cuticle structure of *acs-20* mutants, ultrathin sections were observed under transmission electron microscopy. In *acs-20* mutants, the alae were incompletely extended compared to those of wild-type animals (Fig. 4C and D, black arrows), and unidentified matrix that was rarely observed in wild-type accumulated within the cuticle layers (Fig. 4E and F, arrowheads). In addition, a collagen-like matrix was observed under the alae (Fig. 4D, bold white arrows). Because seam cells are responsible for producing alae [15], we examined the cell fates of hypodermal cells in *acs-20* mutants using a seam cell marker (*scm::gfp*), which localized in the nuclei of seam cells. There were no obvious defects (data not shown). We also examined hypodermal cell integrity using an *ajm-1::gfp* reporter, which is expressed in the apical junctions between epithelial cells. No obvious defect was observed in Hyp7 and the seam cell junction (Fig. 4G, H). Thus, *acs-20* mutants exhibited several abnormalities in the cuticle structure, but not in the epidermal cell fate or cell integrity.

### ACS-20 and ACS-22 Are Involved in VLCFA Incorporation into Sphingomyelin *In Vivo*

**Figure 5:**
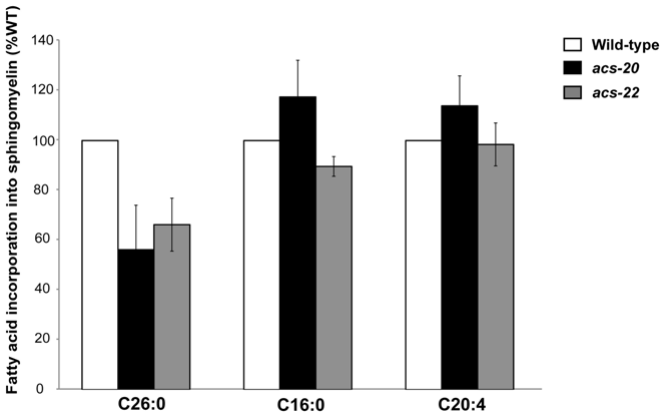
Reduced incorporation of exogenous C26:0 fatty acid into sphingomyelin in *acs-20* and *acs-22* mutants. Incorporation of [^14^C]C26:0, [^14^C]C16:0 and [^14^C]C20:4 into sphingomyelin is expressed as the percentage of the wild-type value. All experiments were performed at 20°C. Bars represent SEMs of two independent experiments.

Finally, we analyzed the incorporation of radio-labeled fatty acids into the lipid fraction of worms. Because VLCFAs are not detected in glycerolipids, but in sphingolipids, in *C. elegans*, as shown in the study of Gerdt *et al.* [16] and Menuz *et al.* [17], we analyzed the incorporation of radio-labeled VLCFA into the sphingomyelin fraction of worms. In this system, free ^14^C-fatty acids are converted to [^14^C]acyl-CoAs by endogenous acyl-CoA synthetases and then incorporated into sphingomyelin. The incorporation of C26:0 fatty acids into sphingomyelin was reduced in *acs-20* and *acs-22* mutant animals (Fig. 5). On the other hand, the incorporation of C16:0 and C20:4 fatty acids was not affected in *acs-20* and *acs-22* mutants (Fig. 5). These data indicate that *acs-20* and *acs-22* are involved in incorporating VLCFA, but not other fatty acids, into the sphingomyelin *in vivo*.

## Discussion

In the present study, we characterized two *C. elegans FATP* genes, revealing that *acs-20* mutants are defective in the cuticle barrier that prevents penetration of small molecules. *acs-22* mutants rarely exhibited a barrier defect, but the *acs-20;acs-22* double mutant showed severely disrupted barrier function, suggesting a redundant function of the genes. We also showed that the barrier defects of *acs-20* and *acs-20;acs-22* mutants are rescued by *acs-20*, *acs-22,* and human *Fatp4* transgenes. These data indicate that *acs-20* and *acs-22* are functional homologues of mammalian *Fatp4*.

In the rescued lines, we observed the reticular expression pattern of human FATP4::Venus protein throughout the cytoplasm, which is compatible with the finding that mammalian FATP4 is localized in the ER [18]. Although several initial studies suggested that FATP4 localizes to the plasma membrane of adipocytes and intestinal epithelial cells and facilitates fatty acid uptake [19], [20], other studies suggest that the protein localizes to the intracellular membrane and functions as an acyl-CoA synthetase [18], [21]. In the present study, we observed the functional expression of *C. elegans* FATPs and human FATP4 in an ER-like pattern rather than a plasma membranous pattern, indicating that FATP localization in the ER is essential for surface barrier function. These findings further suggest that ACS-20, ACS-22, and FATP4 act as acyl-CoA synthetases, but not as fatty acid transporters, for surface barrier function.

The *acs-20* mutants exhibited abnormalities in the cuticle structure. The *C. elegans* cuticle is a highly structured extracellular matrix (ECM) that is composed predominantly of cross-linked collagens, additional insoluble proteins termed cuticlins, and associated glycoproteins and lipids. ECM is synthesized and secreted by underlying epithelial cells, the hypodermis, and seam cells [22]. Although the mammalian skin appears to be largely different from the worm cuticle, some fundamental similarities exist, e.g., in the mammalian stratum corneum, the lipids discharged from the underlying epithelial cells, the keratinocytes, into the intercellular space and are cross-linked with a variety of proteins [23]. Given that *Fatp4*-deficient mice and *acs-20* mutant worms have surprisingly similar defects in the skin and cuticle barrier, respectively, the defects are likely to be caused by common mechanisms between mammals and worms.

We propose two possible causes of the cuticle barrier defect: Given that *acs-20* mutants displayed an unusual accumulation of collagen-like matrix under the alae, one possibility is an alteration of the secretion process of ECM from epithelial cells that results in the barrier defect. Because sphingomyelin is essential in lipid rafts of the plasma membrane [24] and our study showed reduced incorporation of C26:0, but not C16:0 or C20:4, into the sphingomyelin in *acs-20* and *acs-22* mutants, VLCFA-containing sphingomyelin in the epithelial cell membrane may be involved in regulating ECM secretion. Another possibility is that the altered lipid composition in the cuticle affects the cuticle barrier. Ceramide, which is synthesized from sphingomyelin and secreted from epithelial cells, might be involved in cuticle barrier function. Because VLCFA is highly hydrophobic, VLCFA-containing ceramide is likely to contribute to form a physicochemical barrier in the cuticle ECM. In this case, the unusual secretion of collagen-like matrix observed in *acs-20* mutants may compensate for the impaired lipid barrier.

In addition to animals, several lines of evidence indicate that plant long-chain fatty acid acyl-CoA synthetases (LACSs), LACS1 and LACS2, are involved in normal cuticle development and barrier function [25], [26]. The FATP family in animals and the LACS family in plants may have a common role in skin or cuticle development/barrier, although plant LACSs show very low overall similarity with mammal or *C. elegans* FATPs, except for a small stretch of amino acids, which is highly conserved AMP-binding motif in the ACS family. In addition, Tang *et al.* demonstrated that LACS2 mutations enhance susceptibility to *Pseudomonas syringae* but confer resistance to *Botrytis cinerea* [27], suggesting that FATP mutants might have an altered susceptibility to pathogens. Further studies are needed to elucidate the involvement of FATP in *C. elegans* defense mechanisms.

*acs-20* mutants may be valuable for use in drug screening. Recently, *C. elegans* attracted the attention of drug screening and chemical genomics researchers [28], [29]. One problem that remains to be resolved for this type of drug screening, however, is the low accessibility of compounds because the worm cuticle acts as a barrier to small molecule diffusion [30]. *acs-20* mutant animals have an impaired cuticle barrier that results in an increase in small molecule accessibility. Moreover, basic physical parameters such as viability and locomotion are rarely affected in *acs-20* mutants, suggesting its utility in drug screens targeting neurologic disorders and many other diseases.

In summary, ACS-20 and ACS-22 are involved in the surface barrier in *C. elegans*. Our study is the first to show a biologic significance of FATPs in *C. elegans* and reveals that human FATP can substitute for worm FATP4. We propose that the *C. elegans* FATP mutant is a simple animal model for studying fundamental molecular mechanisms underlying the mammalian skin barrier. Future studies using the *C. elegans* model should facilitate further biologic and pharmacologic studies and may lead to therapeutic approaches to human skin diseases.

## Materials and Methods

### Strains

*C. elegans* strains were cultured using standard techniques [31]. The wild-type strain Bristol N2, *kaIs12[col-19::gfp]*, *wIs51[scm::gfp],* and *jcIs1[ajm-1::gfp]* were obtained from the Caenorhabditis Genetics Center. Strains carrying the following mutations were obtained from the trimethylpsoralen/ultraviolet mutagenized library, as described previously [32] and identified by polymerase chain reaction (PCR) amplification with primers spanning the deletion regions: *acs-20(tm3232)IV*, *acs-20(tm3278)IV*, *acs-22(tm3236)X, acs-22(tm3290)X*. The *acs-20* mutants and *acs-22* mutants were backcrossed twice with N2. Primers used for nested PCR screening were as follows; tm3232&tm3278_1^st^ round: 5′- ATCGCGTAGCTGGTAAGCGA-3′, 5′- TTGAGGTTTACTCTGTCGGA-3′, 2^nd^ round: 5′- ACTCGAACAACTGACGCCAT-3′, 5′- ACTACGGAACCAAAGACCCT-3′, tm3236&tm3290_1^st^ round: 5′- CTTGGGTTCACATCGTACGT-3′, 5′- AGCAGTCCTGGAAATACGTC-3′, 2^nd^ round: 5′- GGTTACAAAATGGGCGACGT-3′, 5′- GAGCCGTACAACTCTCCAAT-3′.

### Constructs and Transgenic Lines

The cDNA clones encoding *acs-20* and *acs-22* were obtained by reverse transcription (RT)-PCR from mixed-stage *C. elegans* Bristol N2 cDNA with primers as follows; acs-20_sense: 5′-ATGAAGCTGGAGGAGCTTGT-3′, acs-20_antisense: 5′-TTAAAATGGATAACTTCCCAGTGAG-3′, acs-22_sense: 5′-ATGAGGGAAATGCCGGACAG-3′, acs-22_antisense: 5′-TTAAATGCGATCATAAACACCAGTG-3′. The cDNA clone encoding human *Fatp4* (*hsFatp4*) was amplified from HeLa cell cDNA with primers as follows; hsFatp4_sense: 5′-ATGCTGCTTGGAGCCTCTCT-3′, hsFatp4_antisense: 5′-TCACAGCTTCTCCTCGCCTG-3′. All coding sequences used in the present study were verified by sequencing. To generate *P_acs-20_::egfp*, *P_acs-22_::egfp* and *P_acs-20_::acs-20::egfp* plasmids, the genomic fragments described in Fig. 1B were amplified and cloned 5′ to the EGFP in the pFX_EGFP expression vector [33]. For hypodermis-specific expression of ACS-20, ACS-22, or hsFATP4, the *dpy-7* promoter sequence was used as previously described [34]. The *dpy-7* promoter sequence and amplified cDNAs were cloned in the pFX_venusT expression vector in-frame [33]. To generate transgenic lines, constructs were injected at 100 to 180 ng/µl along with pRF-4*[rol-6d]* or *P_gcy-10_::DsRed* as an injection marker (20-100 ng/µl). The transgenic arrays constructed for this study were; *tmEx1821 [P_acs-20_::egfp*, *rol-6d]*, *tmEx1855 [P_acs-22_::egfp*, *rol-6d], tmEx1920 [P_acs-20_::acs-20::egfp, P_gcy-10_::DsRed], tmEx1955[P_dpy-7_::acs-20:Venus, P_gcy-10_::DsRed], tmEx1983[P_dpy-7_::acs-22:Venus, P_gcy-10_::DsRed], tmEx1995 [P_acs-20_::acs-20::egfp, P_gcy-10_::DsRed], tmEx2071[P_dpy-7_::hsFATP4::Venus, P_gcy-10_::DsRed], tmEx2072[P_dpy-7_::hsFATP4::Venus, P_gcy-10_::DsRed].*

### Measurement of Body Length

We used 1-day-old adult hermaphrodites (synchronized L4-stage larvae were cultured for 24 h at 20°C). Animals were transferred onto a glass slide and paralyzed. Body lengths of animals were measured with a dissecting microscope and a micrometer.

### Nile Red and C1-BODIPY-C12 Staining

Staining experiments were performed as previously described [35]. Nile Red (Molecular Probes, Eugene, OR) was dissolved in acetone to produce a 0.5-mg/ml stock solution and stored at 4°C. C1-BODIPY-C12 (Molecular Probes) was dissolved in dimethylsulfoxide to produce a 5-mM stock solution and stored at −20°C. Stock solutions were freshly diluted in 1x PBS to 1 µg/ml (Nile Red) or 1 µM (C1-BODIPY-C12) and 150 µl of the diluted solution was poured onto Nematode Growth Media plates (6-cm diameter, 8 ml agar) seeded with *E. coli* OP50. Plates were allowed to air-dry before use. Starved worms were cultured on the plates for 3 days before observation.

### Lipid Content Analyses

Phospholipid and triglyceride content: Lipids of synchronized young adult worms were extracted by the method of Bligh and Dyer [36]. The phosphorus content of the total lipids was determined by the method of Bartlett [37]. The triglyceride content was measured using an enzymatic kit (Wako Triglyceride E-test, Wako Pure Chemical Ltd., Osaka, Japan).

### Hoechst Staining, Sensitivity to Hypo-Osmolality

Hoechst staining was performed as described previously [38] except that we used membrane-permeable and cuticle-impermeable Hoechst 33342 (formula weight, 561.93) instead of membrane- and cuticle-impermeable Hoechst 33258 (formula weight, 533.88) to evaluate the cuticle barrier rather than the plasma membrane barrier. Briefly, worms were incubated in M9 buffer containing 1 µg/ml Hoechst 33342 (Wako Pure Chemical Ltd., Osaka, Japan) at room temperature for 15 min, followed by washing several times with M9 buffer. Fluorescence images were captured with the identical parameters (lens and magnifier used, filters, exposure time, resolution) and with an 8-bit pixel depth (256 shades of grey). Fluorescence intensity was examined by processing fluorescence micrographs with ImageJ (Rasband, W.S., US National Institutes of Health, Bethesda, MD, http://rsb.info.nih.gov/ij/). To analyze the fluorescence of stained nuclei without including the auto-fluorescence of the intestine, the tail area was enclosed using a freehand selection tool, excluding the intestinal area. The average pixel intensity was determined. Sensitivity to hypo-osmolality was assayed by placing young adult worms into 20-µl drops of distilled H_2_O and measuring the time until they became rigid. All experiments were performed at 20°C.

### Microscopy

Differential interference contrast and fluorescence images were obtained using a BX51 microscope equipped with a DP30BW CCD camera (Olympus Optical Co., Ltd, Tokyo, Japan).

### Transmission Electron Microscopy

Transmission electron microscopy was performed by the Hanaichi Ultrastructure Research Institute Co. (Okazaki, Japan). Wild-type or mutant larvae were fixed with 4% paraformaldehyde and 1% glutaraldehyde, 1% paraformaldehyde in 100 mM cacodylate buffer for 1 h each at room temperature, followed by further fixation with 2% paraformaldehyde and 2% glutaraldehyde in 100 mM cacodylate buffer for 1 day and an overnight wash in 100 mM cacodylate buffer at 4°C. They were then postfixed for 24 h with 2% osmium tetroxide in 100 mM cacodylate buffer, followed by dehydration and infiltration with a resin Q651 (Mitsui Chemicals, Tokyo, Japan). Ultrathin sections of the surface area were analyzed using an electron microscope (JOEL JEM-200EX, Akishima, Japan).

### *In Vivo* Incorporation of Exogenous Fatty Acids

Synchronized first-stage larvae (approximately 6000 animals) were cultured with 1 µCi of ^14^C-labeled palmitic acid, arachidonic acid, and cerotic acid (C16:0, C20:4, and C26:0, respectively) on *C. elegans* growth medium plates at 20°C until they reached the young adult stage. Lipids were extracted by the previously described methods [36] and separated by one-dimensional thin layer chromotography on silica gel 60 plates (Merck, NJ) in chloroform/methylacetate/1-propanol/methanol/0.25% KCl (25/25/25/10/9, v/v). Incorporation of ^14^C-labeled fatty acids into sphingomyelin was calculated based on the percentages of the whole radioactivity incorporated into the total lipids.
